# Diverse human extracellular RNAs are widely detected in human plasma

**DOI:** 10.1038/ncomms11106

**Published:** 2016-04-26

**Authors:** Jane E. Freedman, Mark Gerstein, Eric Mick, Joel Rozowsky, Daniel Levy, Robert Kitchen, Saumya Das, Ravi Shah, Kirsty Danielson, Lea Beaulieu, Fabio C. P. Navarro, Yaoyu Wang, Timur R. Galeev, Alex Holman, Raymond Y. Kwong, Venkatesh Murthy, Selim E. Tanriverdi, Milka Koupenova, Ekaterina Mikhalev, Kahraman Tanriverdi

**Affiliations:** 1 University of Massachusetts Medical School, Department of Medicine, Division of Cardiovascular Medicine, Worcester, Massachusetts 01605, USA; 2 Yale University Medical School, Computational Biology and Bioinformatics Program, New Haven, Connecticut 06520, USA; 3 University of Massachusetts Medical School, Department of Quantitative Health Sciences, Worcester, Massachusetts 01605, USA; 4 The Framingham Heart Study, Framingham, Massachusetts 01702, USA; 5 Population Sciences Branch, National Heart, Lung, and Blood Institute, National Institutes of Health, Bethesda, Maryland 20824, USA; 6 Cardiovascular Institute, Beth Israel Deaconess Medical Center, Boston, Massachusetts 02215, USA; 7 Center for Cancer Computational Biology, Dana Farber Cancer Institute, Boston, Massachusetts 02215, USA; 8 Cardiovascular Division, Department of Medicine, Brigham and Women's Hospital, Boston, Massachusetts 02115, USA; 9 University of Michigan, Ann Arbor, Michigan 48409, USA

## Abstract

There is growing appreciation for the importance of non-protein-coding genes in development and disease. Although much is known about microRNAs, limitations in bioinformatic analyses of RNA sequencing have precluded broad assessment of other forms of small-RNAs in humans. By analysing sequencing data from plasma-derived RNA from 40 individuals, here we identified over a thousand human extracellular RNAs including microRNAs, piwi-interacting RNA (piRNA), and small nucleolar RNAs. Using a targeted quantitative PCR with reverse transcription approach in an additional 2,763 individuals, we characterized almost 500 of the most abundant extracellular transcripts including microRNAs, piRNAs and small nucleolar RNAs. The presence in plasma of many non-microRNA small-RNAs was confirmed in an independent cohort. We present comprehensive data to demonstrate the broad and consistent detection of diverse classes of circulating non-cellular small-RNAs from a large population.

## Abstract (web summary)

Extracellular miRNAs are present in a variety of bodily fluids. Here, Freedman *et al*. analysed plasma-derived RNA by RNA-seq from 40 people followed by targeted RT-qPCR in an additional 2,763 people, and report over 1,000 extracellular RNAs including microRNAs, piwi-interacting RNA and small nucleolar RNAs.

## Graphical abstract

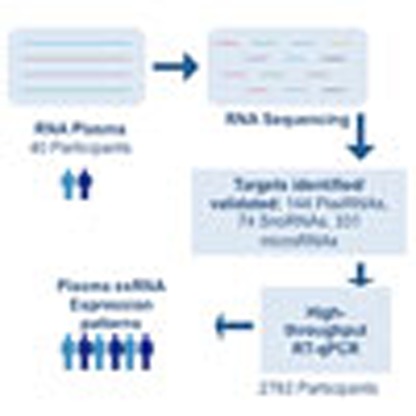

The discovery of small-RNAs, including microRNAs (miRNAs) has dramatically altered our understanding of the regulation of gene expression^1^. Extracellular miRNAs are present in a variety of bodily fluids including plasma, urine and saliva, and these molecules are notably stable and resist degradation despite the presence of RNase^2^,^3^. The discovery of stable RNA outside of cells has transformed our understanding of the role RNA may play in cell-to-cell communication and other complex processes. In addition, expression of cellular and extracellular miRNAs has also been associated with a wide variety of diseases^4^,^5^,^6^. Although miRNAs are observed in the extracellular space, little is known about the extracellular presence of other common varieties of small human RNAs such as piwi-interacting RNA (piRNAs) and small nucleolar RNAs (snoRNAs), known to be key components of molecular interactions and gene regulation in eukaryotes. piRNAs are a distinct class of 26–31 nucleotide-long RNAs produced by a Dicer-independent mechanism that may have function beyond transposon silencing and snoRNAs primarily guide chemical modifications of other RNAs, however, their expression in human populations is unknown.

Based on limited RNA sequencing (RNAseq) data, it has been recently suggested that human extracellular spaces may contain a far broader array of RNAs^7^,^8^, but their rapid simultaneous identification has been hampered by bioinformatic limitations. After identification of unbiased human small-RNAs using newer RNAseq bioinformatics pipelines, we performed a secondary study in Framingham Heart Study (FHS) participants revealing the range of detectable extracellular RNAs in a large human population. These findings were confirmed using an independent cohort from the Extracellular RNA Communication Consortium. Previous unbiased studies of exRNAs have studied very small numbers or pooled samples for the purpose of identifying a class of small-RNAs^9^. Larger human studies have shown the distribution of exRNAs, with a focus exclusively on miRNAs. Most previous studies, however, have been limited by measuring only targeted miRNAs in higher numbers of individuals or more broad measurements in very small numbers of individuals. Our study presents the largest description of plasma-based miRNAs and the first description of the broad variety of extracellular (non-miRNA) small-RNAs in a large population.

Here, we analysed sequencing data from plasma-derived RNA from 40 individuals and identified over a thousand human extracellular RNAs including microRNAs, piRNA, and snoRNAs. Using a targeted quantitative PCR with reverse transcription (RT-qPCR) approach in an additional 2,763 individuals, we characterized almost 500 of the most abundant extracellular transcripts. The presence in plasma of many non-microRNA, small-RNAs was confirmed in an independent cohort. These findings show that diverse classes of circulating non-cellular small-RNAs, beyond miRNAs, are consistently present in plasma from multiple human populations.

## Results

### Gene expression in human plasma by RNAseq

**Figure 1:**
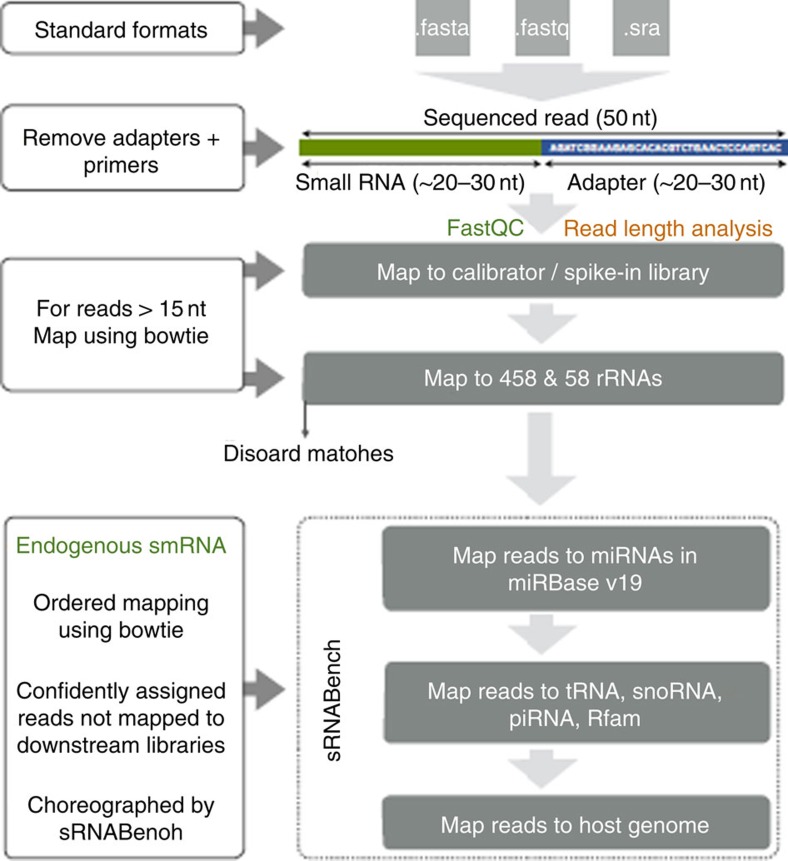
Sequencing data analysis using the Genboree-sequencing pipeline. Small-RNAseq reads were processed and quantified using the exceRpt tool available on the Genboree Workbench. ExceRpt incorporates several modifications to existing analysis methods used to assess cytosolic miRNAs that specifically address experimental issues pertinent to exRNA profiling, such as variable contamination of ribosomal RNAs, and the presence of endogenous non-miRNA small-RNAs. Briefly, the software processes each sample independently through a casade of read-alignment steps designed to remove likely contaminants and endogenous sequences before aligning to exogenous miRNAs.

To determine the broadest number of exRNAs in human plasma, we performed RNAseq on 40 stored plasma samples from FHS participants (Offspring Cohort Exam 8, demographics in Supplementary Table 1). The FHS is a community-based, prospective study with participants undergoing an examination every ∼4–8 years. Study participants have been densely phenotyped over multiple prior examinations. RNA was isolated from the 40 plasma samples and RNAseq performed to determine the presence and abundance of human small-RNAs including miRNAs and other small exRNAs. On the basis of comparison of RNA plasma isolation kits, the Exiqon Biofluid kit was chosen for sample isolation before RNAseq and RT-qPCR analysis (Supplementary Fig. 1). The 40 study participants had a mean age of 68±8 years of age, with an average body mass index (BMI) of 28.2±4.3 mg kg^−2^., 50% of participants were female, and 50% had a prevalent cardiovascular disease (CVD). Sequencing was performed using an ion proton platform (see overview in Supplementary Fig. 1B). Sequencing data were processed in the Genboree-sequencing pipeline (Fig. 1) and comparative analyses were performed.

Analyses of RNAseq data from 40 human plasma samples identified a total of 1,192 small human RNAs above 1^3^ reads per million (RPM; most abundantly expressed RNAs are listed in Supplementary Table 1; a summary list is provided in Supplementary Table 2A,B). As expected, miRNAs were widely and abundantly expressed. While there were many miRNAs identified, miR-223-3p and miR-451a were the most abundant in plasma and accounted for 47% of all plasma-sequencing readings. These specific miRNAs have been found to be most abundant in previous studies^10^,^11^. While the dominance of these miRNAs could have resulted in false negative findings for some low-abundant targets, an extraordinary range of non-miRNA small-RNAs was identified. In addition, patterns of relative expression do not suggest that red blood cell lysis was the cause of the increased expression. For miR-23a-miR-451, increased delta quantification cyle (Cq) values <5 would suggest little or no haemolysis and >7 would be indicative of significant haemolysis. This assumption is based on a previous report showing that miR-23a is relatively stable in plasma and not affected by haemolysis^12^.

For our samples, the delta Cq value of miR-23a and miR-451 were <5.0 in 2,179 out of 2,763 samples. Four-hundred thirty-nine samples had values between 5.0 and 6.99, 9 samples did not express miR451, 24 samples did not expressed miR-23a and 88 samples did not expressed either miRNA. Only 24 samples had a value between 7.0 and 8.42 representing less than 1% of all samples. Although not previously known to be widely present in the circulation, we identified 144 small-RNAs that mapped to piRNAs. piRNAs differ from miRNAs in size (26–31 nt rather than 21–24 nt) and are known to form RNA–protein complexes by interacting with piwi proteins that regulate either epigenetic or post-transcriptional gene silencing^13^. We also found 74 snoRNAs and although limited information is available in regard to their expression in human tissues, they have been previously reported in studies using RNAseq^14^.

### Validation of piRNAs and snoRNAs in an independent cohort

To provide external validation of identified piRNAs and snoRNAs, we utilized a cohort that had undergone RNAseq of plasma samples using independent RNA isolation, RNAseq (Illumina), and analyses (see Supplementary Methods for details). This cohort, based at the Beth Israel Deaconess Medical Center, consisted of normal individuals (*n*=3; 1 female; mean age 25.7±9 years) and post-myocardial infarction (MI) patients who had plasma collected within 4 weeks after presentation (*n*=40, 20% female, mean BMI 28.9±5.2 kg m^−2^). Patients studied after MI were derived from a completed, multi-center study of post-MI cardiac structural changes (PROSPECT-CMR study; ClinicalTrials.gov identifier: NCT00729430). Patients in this study were enrolled 14–28 days after an acute MI, with subsequent randomization to fish-oil therapy and follow-up at 6 months after randomization. A subset of plasma samples from the baseline (before randomization) study visit was used for RNA sequencing.

**Figure 2:**
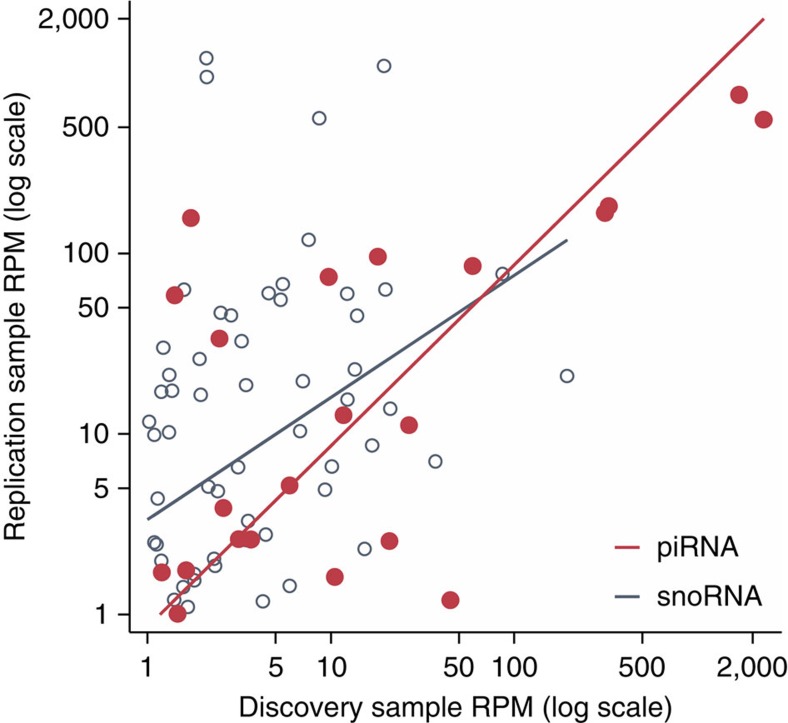
Overlapping expression of piRNAs and snoRNAs between post-MI and FHS Cohorts. Using the same detection threshold as in our discovery phase (average RPM≥1 K combined), the replication sample detected 111 piRNAs and 164 snoRNAs with statistically significant correlation between the RPM values for snoRNAs (Spearmans *ρ*=0.32, *P*=0.02) and piRNAs (Spearman's *ρ*=0.53, *P*=0.01) between the FHS and BIDMC cohorts.

Using the same detection threshold as in our discovery phase (average reads per million (RPM)≥1K combined), the replication sample detected 111 piRNAs and 164 snoRNAs. Of note, 15% (22 of 144) piRNAs and 76% (56 out of 74) snoRNAs detected at ≥1K RPM in the FHS cohort were also detected in the replication sample. Overlapping exRNAs were similarly abundant in each cohort (Fig. 2) with statistically significant correlation between the RPM values for snoRNAs (Spearmans *ρ*=0.32, *P*=0.02) and—to a greater extent—piRNAs (Spearman's *ρ*=0.53, *P*=0.01). Together, these data suggest the validity of the presence of multiple sequences consistent with piRNA and snoRNA species in human plasma.

### exRNAs in 2,763 individuals

From the RNAseq data, we developed a list of observed exRNAs (*n*=485) that we targeted using high-throughput RT-qPCR (Fluidigm BioMark system, Methods and Supplementary Fig. 1C)^15^,^16^ in 2,763 additional participants from the FHS. To ensure reproducibility, performance testing was conducted. cDNA replicate and preamplification replicates had associations typically above 0.9. This large unselected sample of FHS study participants had a mean age of 67±9 years of age, was 54% (*n*=1,499) female, had an average BMI of 28.3±5.4 mg kg^−2^, had a 23% prevalence of CVD. After removing poorly performing assays we measured the expression of 331 human miRNAs, 97 human piRNAs, and 43 human snoRNAs. Because piRNAs have a 3′ modification (2′-*O*-methylation), we utilized a modified approach to be able to analyse them by RT-qPCR.

**Figure 3:**
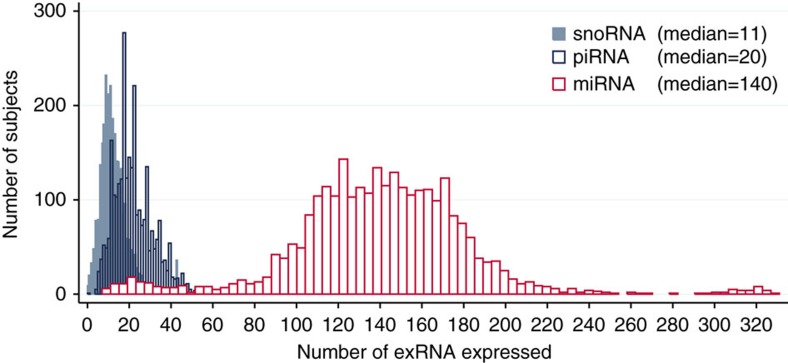
Distribution of plasma exRNA expression in 2,763 Framingham Heart Study participants. From the RNAseq data we developed a list of observed exRNAs to target using high-throughput RT-qPCR (Fluidigm BioMark system). The expression of 331 human miRNAs, 97 human piRNAs, and 43 human snoRNAs were measured (Cq<23). Because piRNAs have a 3′ modification (2′-*O*-methylation), we utilized a modified RT-qPCR approach. The abundance and distribution of each type of small-RNA in the FHS cohort is illustrated in Figure 3 and shows that 50% of the FHS cohort expressed at least 11 snoRNAs and 20 piRNAs in plasma. These results demonstrate considerable variability in terms of number of exRNAs detected in plasma in each participant as well as the overall percent detected.

**Figure 4:**
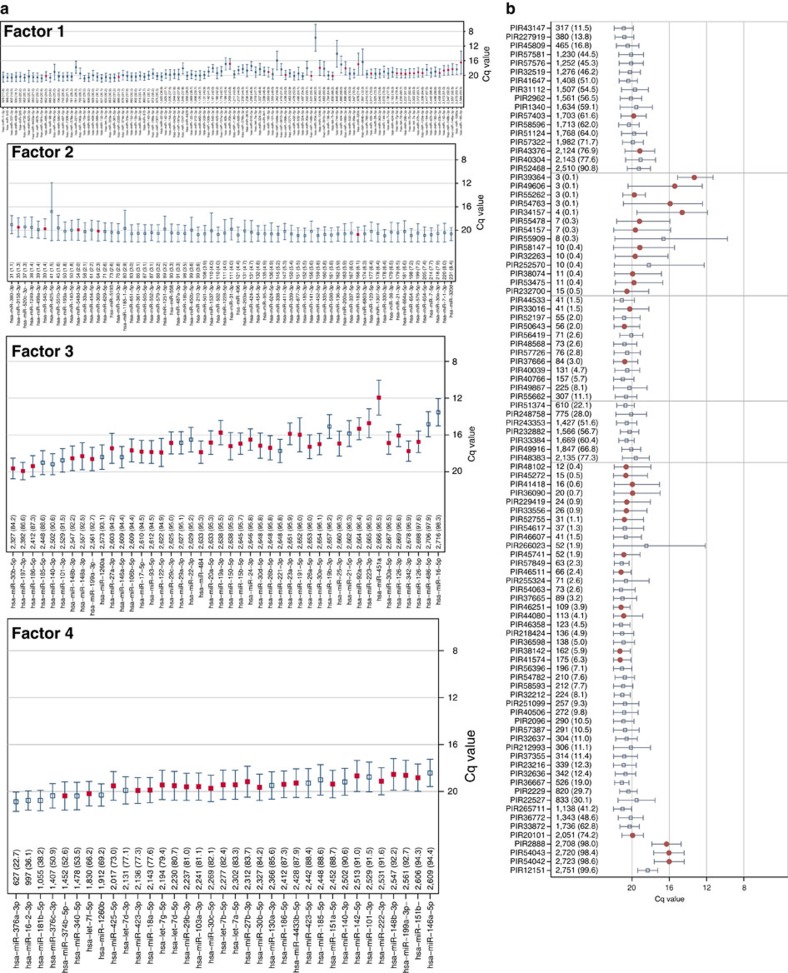
Measurement of plasma miRNAs and piRNAs by RT-qPCR in 2,763 FHS participants. The prevalence and quantity of expression for each miRNA (**a**) and piRNA (**b**) are measured. RNAs are sorted from least to most expressed with the *N* and per cent expressed (that is, Cq<23). The quantification cycle (Cq) axis is sorted in reverse order so that expression increases from bottom to top (**a**) or from left to right (**b**) along the axis. The mean (±s.d.) Cq values are depicted with the horizontal capped lines and small squares (**a**,**b**) or dots (**b**) for each RNA. Light horizontal grey gridlines stratify RNAs by per cent of expression at <10% and >90%. Red squares or dots are used for statistically significant associations for age (*P*<0.05 after adjusting for multiplicity using the FDR) and navy squares are used for RNAs not meeting statistical significance. All supporting values included age and sex of participants are included in Supplementary Tables 1–3 and Supplementary Data 1–3.

The abundance and distribution of each type of small-RNA in the FHS cohort is illustrated in Fig. 3. While there was clearly a greater number of miRNAs expressed in plasma, we found that 50% of the FHS participants had at least 11 snoRNAs and 20 piRNAs detectable in plasma (Fig. 3). These results demonstrate considerable variability in terms of number of exRNAs detected in plasma in each participant as well as the overall per cent detected (Fig. 3). Out of the 331 human miRNAs that were measured by RT-qPCR (Fig. 4), expression ranged from a Cq value of 12 (abundant) to 21 (lower abundance) with most Cq values in the 18–20 range. More abundant miRNAs such as hsa-miR-486-5p (Cq 14.8) and has-miR-451a (Cq 12) were expressed in almost all participants. However, many lower abundance human miRNAs had broad expression as well. In addition to a wealth of data related to miRNA expression, we found other human exRNAs that were present broadly within the study sample. This is consistent with sequence analysis from a single human plasma sample that revealed a diverse collection of exosomal RNA species among which miRNAs were the most abundant, making up over 42% of all raw reads^14^.

**Figure 5:**
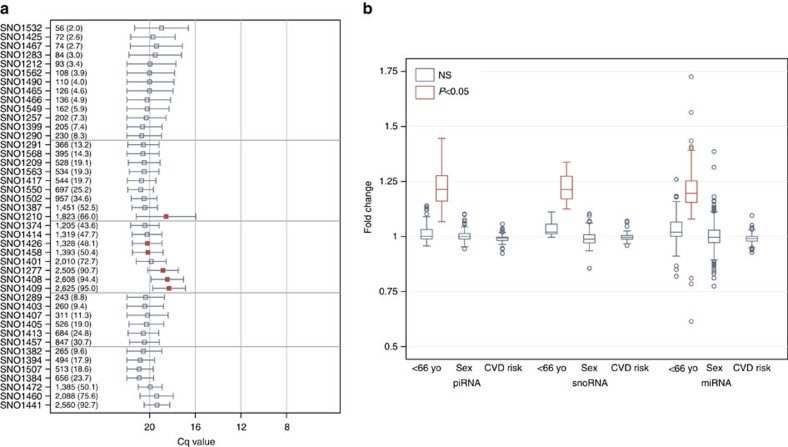
Measurement of plasma snoRNAs and association with age and sex by RT-qPCR in 2,763 FHS participants. (**a**) The prevalence and quantity of expression for each snoRNA are measured. RNAs are sorted from least to most expressed with the *N* and per cent expressed (that is, Cq<23). The quantification cycle (Cq) axis is sorted in reverse order so that expression increases from left to right along the axis. The mean (±s.d.) Cq values are depicted with the horizontal capped lines and small squares for each RNA. Light horizontal grey gridlines stratify RNAs by per cent of expression at <10% and >90%. (**b**) Range of fold-change values associated with age (median split at 66.5 years), sex and cardiovascular risk using the Framingham Risk Score. Red squares or boxes are used for statistically significant associations (*P*<0.05 after adjusting for multiplicity using the FDR) and navy squares and/or boxes are used for RNAs not meeting statistical significance. There were no statistically significant associations with sex for any of the RNAs assessed. In contrast, 39% (*n*=133) of miRNAs, 13% (*n*=13) of piRNAs and 21% (*n*=9) of snoRNAs were associated with age. As shown in Figure 4b and 5a, all piRNAs and snoRNAs and all but three of the miRNAs showed increased expression in subjects <66.5 years of age. All supporting values included age and sex of participants are included in Supplementary Tables 1–3 and Supplementary Data 1–3.

Examination of other human exRNAs revealed highly variable abundance of piRNAs and snoRNAs (Fig. 4b and 5a). Out of the 97 human piRNAs, abundance ranged from Cq values of 13 to 21, although several of the most abundant piRNAs were present in only a few individuals. Nine piRNAs were present in over 2,000 participants. Compared with the human miRNA data, this is the first study to show piRNAs that were ubiquitously present in plasma as well as others that were present in subsets of the study population. For the 43 measured snoRNAs (Fig. 5a), presence was generally lower as compared with the piRNAs (Cq values of 18–20). Numbers of participants with detectable specific snoRNAs ranged from 56 to 2,625. While several piRNAs were present in fewer than 50 individuals, this was not the case for snoRNAs.

### Confirmation of piRNAs

We identified sequences mapping to piRNAs based on unbiased RNAseq, and confirmed this finding in a second independent cohort, and further confirmed it by extensive RT-qPCR. In addition, we performed two distinct reverse transcriptase experiments to show that piRNA RT-qPCR analyses are specific to piRNA 3′modifications. Analyses of cDNAs made for miRNAs and snoRNAs were compared with piRNA assays by RT-qPCR and showed that our cDNA reactions for piRNAs were specific to the piRNA 3′ methylation modification. In addition, plasma RNA samples were treated with sodium periodate or non-treated (nuclease-free water) followed by RNA isolation. Sodium periodate oxidizes the RNA but plant miRNAs and piRNAs are protected from oxidization due to their 3′ methylation. We performed RT-qPCR analyses using piRNA and miRNA assays. The piRNA assays performed equally well in both sodium periodate treated and non-treated RNA samples; but miRNA assays only showed expression for non-treated RNA samples. These findings are consistent with select RNA targets (that is, piRNAs) containing 2′-*O* methylation modifications on the 3′-end protecting from oxidization.

### Association of exRNAs with phenotype

Participants in the FHS Offspring Cohort (at Exam 8) were primarily older with a mean age of 67 and were 54% female (Supplementary Table 1). We compared the expression of each exRNA in older versus younger subjects stratified by the sample median of 66.5 years and in males versus females. There were few exRNAs associated with sex, but several miRNAs, piRNAs and snoRNAs were significantly less expressed in older subjects (Fig. 5b; full results available in Supplementary Data 2). The average 10-year risk for CVD using the FHS prediction model among CVD-free individuals 30–74 years of age (*N*=1,822) was 14±10% (range 0.9–72%). We did not find any exRNA associated with FHS risk score calculated from an individual's age, sex, diabetes status, smoking status, treated or untreated systolic blood pressure and lipid levels (Fig. 5b).

### Factor analysis of exRNAs in 2,763 individuals

We conducted an exploratory principal factor analyses of the miRNAs, piRNAs and snoRNAs in an effort to identify correlated clusters of exRNAs the FHS cohort (full results are available in Supplementary Table 4). For each exRNA type, inspection of the screenplot of eigenvalues suggested that a four-factor solution best represented the factor structure. Descriptive statistics characterizing factor membership are presented in Table 1. Out of the 331 miRNAs, 100 did not load on at least one of the first four factors. The first miRNA factor was comprised of 108 common and uncommon miRNAs (detection rates ranging from 11.9 to 93.5%). The second factor was comprised of 67 uncommonly detected (<8.4% of individuals) miRNAs, while the third was comprised of 40 very commonly detected (>84.2% of individuals) miRNAs. The factors identified among the piRNAs and snoRNAs were similarly defined by the detection rate (see Table 1).

**Figure 6:**
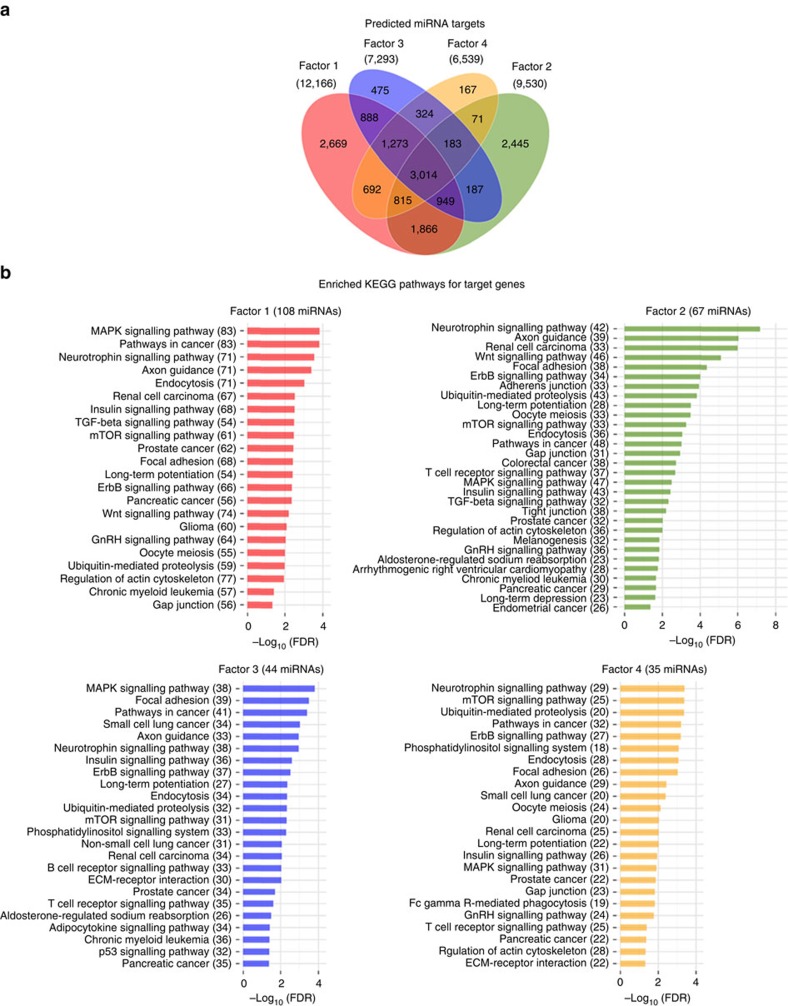
MicroRNA target prediction and pathway enrichment analysis. (**a**) Venn diagram showing the overlaps of the predicted sets of unique mRNAs targeted by miRNAs for each factor. (**b**) Significantly (Benjamini Hochberg corrected *P* value<0.05) enriched KEGG pathways for target genes for factors 1–4. The number of miRNA involved in targeting at least one gene in a given pathway are shown in parentheses.

In an attempt to further characterize these factors we identified the potential targets of the miRNAs loading on each factor using the miRDB Version 5.0 target predictions^17^ available at (http://mirdb.org/miRDB/download.html). After limiting targets to those target prediction scores of at least 80 we found that the Factor-1 miRNAs potentially targeted 23,240 genes. We also found, that these predictions demonstrate some overlap between the factor sets (Fig. 6a). Lastly, we performed KEGG pathway^18^ enrichment analysis of genes targeted by miRNAs in factors 1–4 relative to all target genes in miRDB (Fig. 6b). We found that several pathways showed significant enrichment and overlap such as MAP kinase signalling pathways and those associated with cancer.

### exRNA expression in extracellular and cellular blood sources

To determine the relative cellular/extracellular distribution of the miRNAs, piRNAs and snoRNAs measured in this large population, we performed additional experiments measuring isolated cells, plasma and exosome exRNA expression. Because of the large number of samples (six donors, eight sample types), we performed full exRNA analyses by RT-qPCR using the same methodologies as described for the human samples. Each blood component—plasma, exosomes, platelets, peripheral blood mononuclear cells (PBMC)s, RBCs, T Cells, B Cells, and neutrophils—was isolated as described in the Methods from six healthy donors and flow cytometry was performed to verify the cell type isolated and to identify any potential contaminants or expression of potential cells of origin. For plasma, the sample had minor contamination (from other cell types, Supplementary Fig. 2) but this was, as expected, variable based on the specific sample and could be cellular fragments that were not cleared during centrifugation vs markers of the cell of origin. Thus, it cannot be determined whether these markers are representative of the cells of origin for the exosome vs contamination during preparation. This is a limitation of exRNA studies. For exosomes, the sample was positive for CD9 (an exosome marker; *P*<0.001 by least-squares regression models) and negative for platelets. For platelets, the sample was positive for CD41 (a platelet marker; *P*<0.001) and <5% positive for all other cell markers, except RBCs (11.1%±0.95) and neutrophils (11.8%±2.22). Abundance of each class of exRNAs relative to plasma is shown in Table 2. For piRNAs and snoRNAs, while many were found to be non-significantly different, some cellular forms had increased abundance as compared with plasma. Interestingly, for exosomes, most were not significantly different or decreased (‘upregulated in plasma') as compared with plasma. For miRNAs, few were increased in plasma relative to cells/exosomes, reflecting the separation methods used.

## Discussion

This study is the first systematic description of extracellular non-miRNA small-RNAs in a large sample size of humans and it is the largest data set of exRNAs from human samples. Previous studies of plasma RNAs have focused on the detection of miRNAs, essential exRNAs that regulate the expression of messenger RNAs^19^,^20^. Their abundance and ubiquity and their known bidirectional transport between cells and plasma is important as they are evolutionarily widespread, and participate in a wide range of genetic regulatory pathways^1^. By regulating cell fate choices and transitions between pluripotency and differentiation, miRNAs help to orchestrate developmental events and play a role in tissue homoeostasis that may be important for disease^21^,^22^,^23^. Previous studies have shown that cellular circulating transcripts are associated with CVD and its risk factors^4^,^5^,^15^,^24^,^25^,^26^,^27^,^28^ and future studies utilizing this data set can begin to explore the role of piRNAs and snoRNAs in health and disease. Although the source of circulating exRNA remains unclear, and concerns about cellular contamination vs exosomal release remain, the cellular secretion of unique miRNAs, piRNAs and snoRNAs suggests process specificity^29^,^30^,^31^,^32^. While a strength of our study is that the large numbers allow greater confidence if potential sample preparation contamination occurred, this study, as with all plasma exRNA studies, cannot definitely conclude that all measured circulating exRNAs are due to release *in vivo*. Consistent with a previous study of miRNAs^33^, we found many associations with age and extended these findings to include snoRNAs and piRNAs. In addition, using a large population allowed for factor analyses demonstrating enrichment of specific pathways, however, it also highlights the complexity of using these data for target prediction.

While our findings are consistent with previous studies demonstrating the presence of numerous miRNAs in plasma, this is the first study to report widespread presence of non-miRNA small-RNAs in plasma. The ability to broadly detect non-miRNAs was partially due to the National Institute of Health (NIH) Common Fund strategic planning process established a new program to study extracellular RNAs (exRNAs). One goal of this program was to develop a catalog of exRNAs in human body fluids to facilitate investigation of their roles in health and disease. A crucial objective of this program was to develop bioinformatic tools and pipelines to assist in the broad identification of exRNAs from RNAseq. As part of the Extracellular RNA Communication Consortium, using these recently developed tools, we were able to broadly identify a wide range of extracellular small non-protein-coding RNAs using human plasma. Despite finding numerous piRNAs and snoRNAs in the circulation, their significance to biological processes or disease is unknown. The observation that nonhuman exRNAs are widely, albeit sporadically, present in human plasma is novel and noteworthy as is the striking consistency between separate human studies from diverse populations and utilizing different methods of RNA isolation and RNAseq. Fuller analyses in regards to exact levels of expression would need to be conducted in another large comparable cohort.

Recently, functional roles for piRNA beyond transposon silencing have emerged including regulation of mRNA. piRNAs are a class of small-RNAs that are likely to have existed since the origin of metazoans. Piwi-associated RNAs, as compared with miRNAs, are a distinct class of 26–31 nucleotide-long RNAs produced by a Dicer-independent mechanism. They are associated with Piwi-class Argonaute proteins. Studies in flies, mice and other species implicate these piRNAs in the regulation of germ line development, the silencing of selfish DNA elements, and maintaining germ line DNA integrity. While piRNAs map to unique sites in the human genome, the role of piRNAs in human plasma remains to be elucidated although the association of several with age is intriguing. Also fascinating is the observation of homology between the FHS and validation cohorts (15%), as piRNAs are the largest class of small noncoding RNA molecules expressed in animal cells and it is thought that there are many hundreds of thousands distinct piRNA species. Small-RNA sequencing has been performed in extensive mouse and rhesus macaque samples and showed the presence of piRNA, displaying all known characteristics of piRNA in multiple somatic tissues, suggesting that they may play a role outside of the germ line^34^. They have also been identified in limited bovine samples from whole blood and plasma^35^.

We also found 74 snoRNAs in plasma and there is a paucity of information in regards to their expression in human tissue. Although they have been previously reported by RNAseq^14^, their role in the circulation is currently not clear. Limited numbers of snoRNAs have been shown in lung tissue and plasma from patients with non-small cell lung cancer^36^ and in a small cohort of patients with multiple myeloma^37^. The remarkable consistency (over 50%) between the identifiable snoRNAs from the FHS cohort with the confirmation cohort is striking. However, further studies will be required to determine the reason for this specific pattern of expression.

There are limitations to this study including an older population and a lack of ethnic diversity. However, future studies as part of this consortium will process samples from several ethnically and racially diverse groups as well as younger populations. Our exRNA profiling was performed from plasma samples and, as such, does not provide information about exosomal vs protein-bound RNAs in the large population study. The data suggest that there is a decrease in non-exosomal fractions compared with exosomes but we do not know, based on these experiments, which specific cells the exosomes or protein-bound exRNAs are derived. As expected, the content in the plasma is decreased as compared with the nucleated cells. In addition, our identification of piRNAs is limited in that these are based on sequences that specifically map to piRNAs and we have not established functionality. However, we performed several experiments to establish that targets found as mapping to piRNAs have 3′modifications. Analyses of cDNAs made for miRNAs and snoRNAs with piRNA assays by RT-qPCR, showed that our cDNA reactions for piRNAs were also specific to the piRNA 3′ methylation modification. Additionally, we performed oxidation experiments using plasma exRNAs and show piRNA, but not miRNA, expression in both oxidized and nonoxidized samples. Lastly, while some participants are missing specific abundant miRNAs and/or piRNAs, we do not see uniformly missing exRNAs in these few participants suggesting that it is related to expression and not artifact.

In summary, a limited number of studies have demonstrated a wide variety of exRNAs by RNAseq in humans, but the presence of non-miRNA small-RNAs has not been previously shown. This expansive study demonstrates widespread, variable expression of novel human small exRNAs. Whether they contribute to cellular homoeostasis or are associated with disease pathobiology is not yet known. With broad patterns of expression, it is likely that these intriguing exRNAs are contributing, not in isolation, but in combination to regulate gene expression and influence cellular functions.

## Methods

### Study population

The FHS is a long-term, ongoing cardiovascular study based in Massachusetts. The FHS offspring study is a community-based, prospective study of general disease as well as CVD and its risk factors. The study began in 1948 with 5,209 adult subjects from Framingham, and is now on its third generation of participants. Cohorts undergo an examination at the FHS once every ∼4–8 years and have been densely phenotyped over multiple prior examinations with a wide variety of noninvasive tests. Participants in the Offspring Study have been examined every 4–8 years since the 1970s, for eight prior exams. The participants have extensive antecedent clinical data available and informed consent is obtained for each visit.

### Blood sample collection and plasma separation

Blood samples were previously collected at Framingham Heart Study (Framingham, MA, USA) on Offspring exam 8 (March 2005–Jan 2008). Venipuncture was performed on study participants in a supine position after an overnight fast, using standard venipuncture techniques. Blood was collected into blood collection tubes with a liquid buffered sodium citrate additive (0.105 M). Blood collection tubes were centrifuged at 2,500*g* for 22 min at 4 °C. Plasma was separated from the cells and frozen at −80 °C within 90 min of draw.

An aliquot of 170 μl of plasma samples was transferred to our laboratory in March 2014 and stored at −80 °C. Freezers were both generator and CO_2_ backed up.

### RNA isolation from plasma

RNAs was isolated from plasma samples using a miRCURY RNA Isolation Kit—Biofluids (Cat. No: 300112, Exiqon, Denmark). A total of 130 μl of plasma samples were used in each RNA isolation. The kit is designed to isolate RNA from 200 μl of plasma. We replaced the remaining volume with nuclease-free water. Manufactures protocol was followed with one modification that was reducing the elution volume from 50 μl nuclease-free water to 30 μl. After thawing the plasma samples tubes were mixed by inverting. Then plasma samples were centrifuged at 8,000*g* for 5 min. One-hundred thirty microlitre of plasma samples from the top of the tubes transferred into a new 2.0 ml DNA LoBind tubes (Cat. No: 022431048, Eppendorf, Germany) and 70 μl nuclease-Free water add to each sample to complete the volume to 200 μl. Sixty microlitre Lysis Solution BF were added to each sample. Tubes were vortexed for 15 s. Tubes were incubated for 5 min at room temperature. Twenty microlitre Protein Precipitation Solution BF were added into each tube and tubes were vortexed for 15 s. Vortexed tubes were incubated for 1 min at room temperature. Tubes were centrifuged for 3 min at 16,000*g*. Clear supernatants were transferred into a clean 2.0 ml DNA LoBind tubes. Two-hundred seventy microlitres of Isopropanol was added into each tubes and tubes were vortexed for 5 s. Tubes were centrifuged briefly (2–3 s) to collect all liquid at the bottom of the tubes. MicroRNA Mini Spin Columns BFs connected to vacuum manifold called QIAvac 24 Plus (Cat. No: 19413, Qiagen, Germany) by using VacValves (Cat. No: 19408, Qiagen, Germany). Samples were transferred into microRNA Mini Spin Columns BFs using transfer pipettes. Samples were incubated for 2 min at room temp on microRNA Mini Spin Column BFs with open lids. Vacuum pump was turned on until all liquids were passed. Seven hundred microlitre of Wash Solution 2 BF (80 ml Absolute ethanol was added to Wash Solution 2 BF bottle) was added on microRNA Mini Spin Column BFs and vacuum pump was turned on until all liquids were passed. Two-hundred fifty microlitre of Wash Solution 2 BF was added on microRNA Mini Spin Column BFs and vacuum pump was turned on until all liquids were passed. Hundred microlitre Wash Solution 1 BF was added on microRNA Mini Spin Column BFs and vacuum pump was turned on until all liquids were passed. Seven hundred microlitre of Wash Solution 2 BF was added on microRNA Mini Spin Column BFs and vacuum pump was turned on until all liquids were passed. Two-hundred fifty microlitre Wash Solution 2 BF was added on microRNA Mini Spin Column BFs and vacuum pump was turned on until all liquids were passed. MicroRNA Mini Spin Column BFs were transferred to collection tubes (2.0 ml without a lid). Tubes were centrifuged for 2 min at 11,000*g* at room temperature to dry the membranes completely. Centrifuged microRNA Mini Spin Column BFs were transferred into 1.5 ml DNA LoBind tubes (conical bottom tubes) and 30 μl of nuclease-free water added directly onto membranes. Tubes were incubated for 1 min at room temp with lids open. Lids were closed and centrifuged for 1 min at 11,000*g* at room temperature. RNA samples were transferred into the V bottom, snap cap, 0.5 ml micronic tubes in two aliquots (12.5 μl in each) and capped. 2D barcodes on the tubes were recorded and RNA samples were kept at −80 °C. RNA samples randomly analysed on Bioanalyzer 2100 by using RNA 6000 Pico Kit (Agilent Technologies, Santa Clara, CA).

### Standardization of sample volume

Throughout the experiments, we consistently used 3 μl of vacuum concentrated RNA samples (isolated from 130 μl of plasma) for all samples in library creations for small-RNAseq and 5 μl of RNA samples (isolated from 130 μl of plasma) for all of the samples. A fixed volume of plasma from each participant in the RT-qPCR experiment workflow was used and the same volume of RNA elution (30 μl) was used for all isolations keeping these variables constant across all samples. Also, RNA volumes for reverse transcription reactions (5 μl) were constant for each sample. The same volume (3 μl) of 1:5 diluted cDNA were used in each preamplification reaction. Two microlitre of 1:5 diluted preamplified cDNA samples were used in each qPCR experiment. By doing this we minimized the variance from sample to sample for possible variance for the amount of RNA. Another reason we used a fixed volume based approach is the lack of consensus in regards to housekeeping small-RNAs for extracellular compartments such as plasma.

### Library preparation for RNA sequencing

Ion Total RNAseq Kit v2 (Cat. No: 4479789, Life Technologies, USA) was used for creating libraries for sequencing. Manufacturer instructions were followed with a few exceptions as described below. In collaboration with Life Technologies R&D team, we employed their under-development Ion Adapter Mix to improve performance when using low template amounts. A modified bead purification method was also provided by the Life Technologies R&D team.

### Hybridization

On ice, the hybridization master mix was prepared by mixing 2 μl of the Ion Adaptor Mix v2 with 3 μl of Hybridization Solution. Volumes of the reagents for the master mix were adjusted according to the number of RNA samples used. Five microlitre of the master mix was added onto 3 μl of isolated small-RNA sample and the 8 μl mixture was pipetted up and down five times to mix, then briefly centrifuged to collect the liquid at the bottom of the tube. The hybridization reaction was performed in a thermal cycler as follows: 65 °C for 10 min; and 16 °C for 5 min.

### Ligation

Following hybridization, the ligation master mix was prepared on ice by combining 10 μl of 2X Ligation Buffer with 2 μl of Ligation Enzyme Mix. Volumes of the reagents for the master mix were adjusted according to the number of samples used. Twelve microlitre of ligation master mix was added to each 8 μl hybridization mixture, for a total of 20 μl per reaction. The mixture was pipette up and down 10 times to mix, then centrifuged briefly to collect the liquid at the bottom of the tube. The ligation reaction was performed in a thermal cycler at 16 °C for 16 h.

### Reverse transcription

After ligation, the RT master mix was prepared on ice by combining 2 μl of nuclease-free water, 4 μl of 10 × RT Buffer, 2 μl of 2.5 mM dNTP Mix and 8 μl Ion RT Primer v2 for a total volume of 16 μl. Volumes of the reagents for the master mix were adjusted according to the number of samples used. 16 μl of RT master mix and 20 μl of ligation reaction mixture were mixed, pipette up and down and incubated in a thermal cycler with a heated lid at 70 °C for 10 min, then snap-cooled on ice. Four microlitre of 10 × SuperScript III Enzyme Mix were added to each ligated RNA sample and gently mixed, after which the mixture was incubated in a thermal cycler with a heated lid at 42 °C for 30 min.

### First bead purification

The cDNA was them purified using MagMAX beads. Before the addition of the cDNA samples, 5 μl of the beads were put into one well of a 96-well plate for each sample. Then, 250 μl of Binding Solution Concentrate was added to each well containing beads, and the mixture was pipetted up and down 10 times. Sixty microlitre of nuclease-free water were added to each of the 40 μl RT reactions and transferred to one of the wells of the 96-well plate. 275 μl of 100% ethanol were added to each well and mixed by pipetting up and down. Then the plate was placed on the magnetic stand for 5 min, after which the supernatant was removed carefully without disturbing the beads. 150 μl of Wash Solution Concentrate was added onto the beads and incubated for 30 s. While the plate was still on the magnetic stand the Wash Solution Concentrate was removed. Any leftover Wash Solution Concentrate was removed by using a 10 μl pipette. The beads were then air-dried for 2 min and then the plate was removed from the magnetic stand. 12 μl of pre-warmed (37 °C) nuclease-free water was added on beads, mixed by pipetting up and down 10 times and incubated off the magnetic stand for 1 min. The plate was then put back onto the magnetic stand for 1 min. Purified cDNA was removed carefully and transferred into a DNA LoBind 0.5 ml tube.

### Amplification of cDNA

Following the bead purification, the PCR master mix was prepared by combining 45 μl of Platinum PCR SuperMix High Fidelity and 1 μl of Ion Xpress RNA 3′ Barcode Primer for a total volume of 46 μl per sample. Volumes of the reagents for the master mix were adjusted according to the number of samples used. One microlitre of a selected Ion Xpress RNAseq Barcode primer was individually added into each sample tube, after which 6 μl of cDNA samples were added into each tube. Tubes were mixed and thermal cycling performed on ProFlex 3 × 32 block Thermal Cycler (Life Technologies, USA) as follows: one cycle holds at 94 °C for 2 min, followed by two cycles of 94 °C for 30 s, 50 °C for 30 s and 68 °C for 30 s, followed by 18 cycles of 94 °C for 30 s, 62 °C for 30 s and 68 °C for 30 s, followed by a one cycle holding at 68 °C for 5 min.

### Second bead purification

The amplified cDNA samples were purified by using MagMAX beads. Five microlitre of the beads were added to one well of a 96-well plate for each sample. A total of 280 μl of binding solution concentrate were added to each well containing beads, and mixed by pipetting up and down 10 times. Twenty-seven microlitre of nuclease-free water were added to each of the 53 μl amplified cDNA reactions and transferred to one of the wells of the 96-well plate. Two-hundred thirty microlitre of 100% ethanol were added to each well and mixed by pipetting up and down. The plate was then placed on the magnetic stand for 5 min, after which the supernatant was removed carefully without disturbing the beads. One-hundred fifty microlitre of wash solution concentrate were added and incubated for 30 s. While still on the magnetic stand, the wash solution concentrate was removed. Any leftover wash solution concentrate was removed by using 10 μl pipette. The beads were air-dried for 2 min and the plate was removed from the magnetic stand. Ten microlitre of pre-warmed (37 °C) nuclease-free water were added onto the beads, mixed by pipetting up and down 10 times and incubated off the magnetic stand for 1 min. The plate was then moved back on the magnetic stand for 1 min. Then, the supernatant containing Purified Amplified cDNA was removed carefully and transferred into a DNA LoBind 0.5 ml tube. Amplified cDNA samples were analysed on Bioanalyzer 2100 by using High sensitivity DNA Chip (Agilent Technologies, Santa Clara, CA) to quantify the concentration of PCR bands.

### Template preparation for RNA sequencing

The Ion Chef System and Ion PI IC 200 kits were used for template preparation. The entire procedure was automated by the Ion Chef System. At the end of the template preparation, loaded PI Chips (Life Technologies, USA) were ready for sequencing.

### RNA sequencing on ion proton system

Sequencing reactions were performed on the Ion PI Chip Kit v2 BC and Ion Proton System (Life Technologies, USA) by using the Ion PI Sequencing 200 Kit v2 (Life Technologies, USA). Sequencing reads were at maximum 200 nucleotides.

### RNAseq from plasma

The cohort based at the Beth Israel Deaconess Hospital, consisted of normal subjects (*n*=3; 33% female, age 25.7±9 years) and post-MI patients who had plasma collected within 4 weeks after presentation (*n*=40, 20% female, BMI 28.9±5.2 kg m^−2^). Patients studied after MI were derived from a completed, multi-centre study of post-MI cardiac structural changes (PROSPECT-CMR study; ClinicalTrials.gov identifier: NCT00729430). Patients in this study were enrolled 14–28 days after an acute MI, with subsequent randomization to fish-oil therapy and follow-up at 6 months after randomization. A subset of plasma samples from the baseline (before randomization) study visit was used in RNA sequencing for this analysis.

### RNA isolation from plasma

Total RNA was extracted from 1 ml of plasma using the miRCURY RNA extraction kit for Biofluids (Exiqon) with a modified lysis step. Samples were lysed at 50 °C for 20 min with GITC-containing lysis buffer (provided in the kit) and 10 mg ml^−1^ proteinase K in a 0.5% SDS solution (to a final SDS concentration of 0.005%). Following lysis, RNA extraction was performed according to manufacturer's protocol. Resultant RNA was treated with T4 polynucleotide kinase (NEB) and quantified using Quant-iT RiboGreen RNA Assay (Life Technologies) according to manufacturers' protocols.

### Library preparation for RNA sequencing

Small-RNA libraries were constructed using the NEBNext Multiplex Small-RNA Library Prep Set for Illumina (Set 1), with adaptors diluted 1:6 with nuclease-free water to reduce adaptor dimer formation and sequenced on the Illumina HT HiSeq platform.

### Sequencing data analysis using Genboree-sequencing pipeline

Small-RNAseq reads were processed and quantified using the exceRpt tool available on the Genboree Workbench [ http://www.genboree.org/]. ExceRpt incorporates several modifications to existing analysis methods used to assess cytosolic microRNAs (miRNAs) that specifically address experimental issues pertinent to exRNA profiling, such as variable contamination of ribosomal RNAs, the presence of endogenous non-miRNA small-RNAs, and the presence of exogenous small-RNA molecules derived from a variety of plant, bacteria, and viral species. Briefly, the software processes each sample independently through a casade of read-alignment steps designed to remove likely contaminants and endogenous sequences before aligning to exogenous miRNAs:

(1) *3' adapter clipping*. The adapter clipping is required due to the fact that the majority of small-RNAs are shorter in length than the number of nucleotides sequenced. The adapter removal is performed using the FastX software (v.0.0.13; http://hannonlab.cshl.edu/fastx_toolkit/) using the fastx_clipper tool, and the clipping requires identification of at least 7 adapter bases at the 3′ end of a read and that clipped reads must be longer than 15 nt.

(2) Explicit quality control and filtering is performed on the sequencing reads from each sample by removing likely contaminant sequences derived from laboratory or rRNA contamination. First, rReads are aligned, using Bowtie2 (ref. 38), to UniVec, a library of common contaminant sequences maintained by the NCBI, and reads with valid alignments counted, archived and removed from further consideration. Second, reads are aligned, again using Bowtie2, to the human ribosomal RNA (rRNA) precursor sequences (the full 45S, and 5S, and mitochondrial rRNA sequences) as these rRNAs typically constitute the source of most of the confounding contamination in any small-RNAseq experiment, especially those from extracellular preparations. Reads aligned to the rRNAs are counted, archived, and removed from further consideration in subsequent alignment steps. Bowtie2 is used in both of these steps due to its superior alignment speed, tolerance for gapped alignments, and tolerance for low-quality read sequences.

(3) At its core, exceRpt utilizes the sRNAbench software tool, which is itself based on the miRanalyzer tool^39^,^40^ for mapping and profiling small-RNA libraries. On input to sRNAbench, for each sample, identical clipped-read sequences are counted and collapsed to a single entry and reads containing N's are removed. Clipped, collapsed reads are mapped directly to the human genome and pre-miRNA sequences using Bowtie1 (ref. 38), allowing for only a single mismatched base in each alignment. Bowtie1 is used for this in conjunction with a 19 nt seed in order to allow local alignments at the 5′-end of a small-RNA that might exhibit 3′ non-template additions. The miRNA library used for alignment is obtained from miRBase (v21) (ref. 41). The output from this read-alignment, for each sample, is a collection of files containing the alignment outcomes (pre-miRNA and mature-miRNA IDs) for each read, the read-stack covering each pre-miRNA sequence, and the mapping of the pre- and mature-miRNA sequences with respect to the human genome. These alignments are parsed and used in conjunction with a pre-miRNA secondary-structure prediction, via RNA-fold provided in the Vienna software suite^42^,^43^ to enable verification through stability analysis of the various pre-miRNA hairpin sequences.

(4) Following alignment to the provided miRNA library, reads that did not align in any of the previous steps are mapped against a variety of small-RNA libraries including (with no significance to the ordering): tRNAs from gtRNAdb^43^, piRNAs from RNAdb^44^,^45^, snoRNAs from snoRNA-LBME-db^45^ and snRNAs and other RNA sequences from RFam^46^. In both steps 3 and 4 reads were allowed to multi-map to different annotated transcripts and in cases where this occurred, the read was split proportionally based on the number of equally valid alignments.

(5) Those reads that are not mapped in any of the above steps are taken forward to the final stage of the pipeline where they are aligned, again using sRNAbench, to the complete set of annotated plant and virus pre-miRNA sequences in miRBase.

### RNAseq from the FHS offspring 8 participants

Using the small extracellular RNAseq pipeline (exceRpt) described above, each of the 40 small-RNAseq samples from different individuals were processed independently. The output of the pipeline is the expression values in terms of read counts for all annotated human miRNAs, piRNAs, snoRNAs and tRNAs from miRBase v21 for each sample. The expression value for each sample was normalized to units of RPM total mapped reads. Small-RNAs that had an average expression across the 40 samples above RPM >1 K were selected for RT-qPCR analysis across the entire cohort. This results in a total of 1,192 small-RNAs that included 669 human miRNAs, 144 human piRNAs, 74 human snoRNAs and 305 tRNAs. For the confirmation cohorts, differential expression between groups was performed with the sRNAbenchDE tool using RPM normalized by the total number of reads mapped to the genome for the primary alignment of each sRNA species. Differential expression fold-change and *P* values were calculated with the edgeR^47^ package.

### Reverse transcription for human small-RNAs

Specific piRNAs, snoRNAs and miRNAs were chosen directly from the list generated by RNAseq. The most abundant and most commonly expressed exRNAs were selected for measurement by RT-qPCR. RNA samples (2,763) were reverse transcribed by using miScript II RT Kit (Cat. No: 218161, Qiagen, Fredrick, MD, USA) for measuring human miRNAs and snoRNAs. For piRNAs, miScript Plant RT Kit (Cat. No: 218762, Qiagen, Fredrick, MD, USA) was used due to piRNAs' 3′ modifications. Since plant miRNAs have the same modification this kit allowed us to study human piRNAs by RT-qPCR. Manufacturer instructions were followed for both kits.

### Reverse transcription of miRNAs and snoRNAs

For the reverse transcription for miRNAs and snoRNAs the RNA samples were thawed on ice. Reverse transcription master mix was prepared by combining 950 μl of 5 × miScript HiSpec Buffer, 475 μl of 10 × miScript Nucleics Mix, 475 μl of nuclease-free water, and 475 μl of miScript Reverse Transcriptase Mix for a total volume of 2,375 μl, which was enough for four sets of 96 samples. Five microlitre of this mastermix were dispensed into each well of a 96-well PCR plate (Cat. No: AB17500, Bioplastic, Netherland). Five microlitre of RNA samples were then transferred into each well by using Vioflo384 Pipetting System (Integra Biosciences, Hudson, NH, USA) with 96-well head. Plates were sealed with EU optical Wide 8-Cap Strip Mats (Cat. No: B57651, Bioplastic, Netherland), vortexed on a plate shaker for 10 s by pressing with hands and then centrifuged for 1 min at 500*g* in a small-plate centrifuge. The samples were then incubated at 37 °C for 60 min and then at 95 °C for 5 min in the ProFlex 96 block Thermal Cycler (Life Technologies, USA). When the reverse transcription reactions were completed, plates were centrifuged for 1 min, the strip mats covering was removed and 40 μl of nuclease-free water were added into each well by using Vioflo384 Pipetting System (Integra Biosciences, Hudson, NH, USA) with 96-well head. Plates were again sealed with EU optical Wide 8-Cap Strip Mats (Cat. No: B57651, Bioplastic, Netherland), vortexed on a plate shaker for 10 s by pressing with hands and then centrifuged for 1 min at 500*g* in a small-plate centrifuge.

### Ligation of piRNAs

RNA samples were thawed on ice. Ligation master mix was prepared on ice by combining 950 μl of 10 × miScript Ligation Buffer, 950 μl of 10 × miScript Plant Adaptor and 475 μl of nuclease-free water for a total volume of 2,375 μl, which was enough for four sets of 96 samples. Five microlitre of this mixture were dispensed into each well of a 96-well PCR plate (Cat. No: AB17500, Bioplastic, Netherland). Then, 5 μl of RNA samples were transferred into each well by using Vioflo384 Pipetting System (Integra Biosciences, Hudson, NH, USA) with 96-well head. Finally, 10 μl of 2 × miScript Ligation Activator were pipetted onto each well very slowly, due to viscosity, by using Vioflo384 Pipetting System (Integra Biosciences, Hudson, NH, USA) with 96-well head. The final mixture was mixed using Vioflo384 Pipetting System (Integra Biosciences, Hudson, NH, USA) with 96-well head by pipetting up and down 10 times. Plates were sealed with EU optical Wide 8-Cap Strip Mats (Cat. No: B57651, Bioplastic, Netherland) and centrifuged for 1 min at 500*g* in a small-plate centrifuge. Samples were then incubated at 16 °C for 60 min and at then 65 °C for 20 min in ProFlex 96 block Thermal Cycler (Life Technologies, USA).

### Reverse transcription of piRNAs

Reverse transcription master mix was prepared on ice by combining 1,900 μl of 5 × miScript Plant RT Buffer, 950 μl of 10 × miScript Plant RT Nucleics, 4,275 μl of nuclease-free water and 475 μl of miScript Plant Reverse Transcriptase for a total volume of 7,600 μl, which was enough for four sets of 96 samples. 16 μl of this master mix were dispensed into each well of a 96-well PCR plate (Cat. No: AB17500, Bioplastic, Netherland). 4 μl of the Ligation products were transferred into each well by using Vioflo384 Pipetting System (Integra Biosciences, Hudson, NH, USA) with 96-well head. The mixture was mixed using Vioflo384 Pipetting System (Integra Biosciences, Hudson, NH, USA) with 96-well head by pipetting up and down ten times. Plates were then sealed with EU optical Wide 8-Cap Strip Mats (Cat. No: B57651, Bioplastic, Netherland) and centrifuged for 1 min at 500*g* in a small-plate centrifuge. Samples were then incubated at 37 °C for 2 h and then at 95 °C for 5 min at ProFlex 96 block Thermal Cycler (Life Technologies, USA). When reverse transcription reactions were completed the plates were centrifuged for 1 min, the strip mats covering was removed and 80 μl of nuclease-free water were added into each well by using Vioflo384 Pipetting System (Integra Biosciences, Hudson, NH, USA) with 96-well head. Finally, plates were again sealed with EU optical Wide 8-Cap Strip Mats (Cat. No: B57651, Bioplastic, Netherland), vortexed on a plate shaker for 10 s and then centrifuged for 1 min at 500*g* in a small-plate centrifuge.

### Preamplification of cDNAs

miScript Microfluidics PreAMP Kit (Cat. No: 331455, Qiagen, Fredrick, MD, USA) was used for preamplification reactions. Manufacturer instructions were followed with a modification to reduce the preamplification reaction final volume from 25 μl to 15 μl. Preamplification master mix was prepared on ice by combining 1,425 μl of 5 × miScript PreAMP Buffer, 570 μl of HotStarTaq DNA Polymerase, 1,425 μl of miScript PreAMP Primer Mix, 1,995 μl of nuclease-free water and 285 μl of miScript PreAMP Universal Primer (10 μM) for a total volume of 5,700 μl, which was enough for 4 sets of 96 samples. 12 μl of this master mix was dispensed into each well of a 96-well PCR plate (Cat. No: AB17500, Bioplastic, Netherland). Three microlitre of diluted cDNA samples were transferred into each well by using Vioflo384 Pipetting System (Integra Biosciences, Hudson, NH, USA) with 96-well head. Plates were sealed with EU optical Wide 8-Cap Strip Mats (Cat. No: B57651, Bioplastic, Netherland), vortexed on a plate shaker for 10 s and then centrifuged for 1 min at 500*g* in a small-plate centrifuge. The samples were then cycled in a ProFlex 96 block Thermal Cycler (Life Technologies, USA) as follows: one cycle of the PCR initial activation step for 15 min at 95 °C, followed by two cycles of denaturation for 30 s at 94 °C, annealing for 60 s at 60 °C and extension for 60 s at 60 °C, followed by 10 cycles of denaturation for 30 s at 94 °C, and annealing/ extension for 3 min at 60 °C. When preamplification reactions were completed the plates were centrifuged for 1 min, the strip mats covering was removed and 2 μl of SR1 (side reaction reducer=Exonuclease I) was added into each well by using Vioflo384 Pipetting System (Integra Biosciences, Hudson, NH, USA) with 96-well head. Plates were then sealed again with EU optical Wide 8-Cap Strip Mats (Cat. No: B57651, Bioplastic, Netherland), vortexed on a plate shaker for 10 s and then centrifuged for 1 min at 500*g* on a small-plate centrifuge. Samples were then cycled in at ProFlex 96 block Thermal Cycler (Life Technologies, USA) as follows: 37 °C for15 min; and 95 °C for 5 min. When SR1 reactions were completed, the plates were centrifuged for 1 min, the strip mats covering was removed and 58 μl of nuclease-free water was added into each well by using Vioflo384 Pipetting System (Integra Biosciences, Hudson, NH, USA) with 96-well head. Plates were then sealed again with EU optical Wide 8-Cap Strip Mats (Cat. No: B57651, Bioplastic, Netherland), vortexed on a plate shaker for 10 s and then centrifuged for 1 min at 500*g* on a small-plate centrifuge.

### Real-time PCR miRNA profiling

Assay plates and preamplification primer mix preparation was performed according to the miScript Microfluidics Handbook (https://www.qiagen.com/us/resources/resourcedetail?id=34798505-6ff7-459c-8896-2ee777093f6e&lang=en)). Human miRNA Assays were purchased from Qiagen in dried down format. First, assay plates were centrifuged at 1,500*g* for 10 min. Then, 27.5 μl of nuclease-free water was added into each well and incubated at room temperature for 10 min. Plates were vortexed in a plate shaker for 3 min at room temperature. These assay were at 100 μM concentrations. Eighteen microlitre of these assays were taken and combined in a reservoir (43 snoRNA assays were added separately, see below) and 2,106 μl of nuclease-free water was added to make a preamplification primer pool. 14.25 μl of nuclease-free water was added onto the remaining 9.5 μl of 100 μM assays for a total volume of 23.75 μl and final concentration of 40 μM. Equal volume of miScript Microfluidics Universal Primer (40 μM) was added onto these assays for a total volume of 47.5 μl and final assay concentration of 20 μM. Equal volume of the 2X Assay Loading Reagent (Cat. No: 100-7611, Fluidigm, South San Francisco, CA, USA) (47.5 μl) was added onto these assays. The assays were now ready to load into Dynamic Arrays. 28 μl aliquots of these assays were aliquoted in 96-well Piko plates (Cat. No: SPL0960, Fisher Scientific, USA), sealed and stored at −20 °C. snoRNA assays and piRNA assays were custom designed by Qiagen and synthesized primers were delivered at 100 μM concentration in liquid form. These assays were also prepared as described above.

### Dynamic array preparations for qPCR

Control line fluid was injected into appropriate positions on two Dynamic Arrays 96.96 GE and the Dynamic Arrays were placed into IFC Controller HX for priming. While priming was performed (∼20 min), qPCR master mix was prepared on ice by combining 720 μl of Microfluidics qPCR Master Mix (Qiagen), 72 μl of 20 × DNA Binding Dye Sample Loading Reagent (Fluidigm, PN 85000746), and 168.00 μl of nuclease-free water for a total of 960 μl, which was enough for two sets of 96 samples. Four microlitre of this master mix were dispensed into each well of a 96-well PCR plate (Cat. No: AB17500, Bioplastic, Netherland). Two microlitre of diluted preamplified cDNA samples were then transferred into each well using Vioflo384 Pipetting System (Integra Biosciences, Hudson, NH, USA) with 96-well head. Plates were then sealed with EU optical Wide 8-Cap Strip Mats (Cat. No: B57651, Bioplastic, Netherland), vortexed on a plate shaker for 10 s and then centrifuged for 1 min at 500*g* on a small-plate centrifuge. Five microlitreof this mixture were aspirated by using Viaflo 8-channel electronic pipet and dispensed into sample inlets of Dynamic Arrays as 4.75 μl to avoid creating bubbles. 4.75 μl of assays were aspirated by using Viaflo 8-channel electronic pipet and dispensed into sample inlets of Dynamic Arrays as 4.50 μl to avoid creating bubbles. Dynamic Arrays were placed into the IFC Controller HXs and the standard Load script was started (∼90 min). When the loading script was finished, dynamic arrays were loaded into the BioMark reader and cycling was performed as follows: thermal mix for 2 min at 50 °C; 30 min at 70 °C; and 10 min at 25 °C, followed by PCR initial activation step for 10 min at 95 °C, followed by a three-step cycling for 23 cycles of denaturation for 15 s at 94 °C, annealing for 30 s. at 55 °C and extension for 30 s. At 70 °C, followed by performing fluorescence data and collection and dissociation curve analysis. ROX was used as a passive reference dye. Linear derivative and user global setting (Threshold setting 0.002) were used as setting parameters, samples and assays names were entered and basic real-time PCR analysis was performed on BioMark Real-Time PCR Analysis software. Quantification cycle (Cq) values were exported as csv files.

### Poorly performing PCR assays

Poorly performing assays were removed from analysis. Assays that had consistent, nonvariable expression in samples and no-template RT Control samples were removed as this result demonstrates lack of assay specificity for the target. Using these criteria, a total of 15 assays were removed from the initially designed 486 assays.

### Blood cell isolation

*Red blood cells*. Citrated whole blood was centrifuged at 150*g* for 17 min with no brake. After the platelet rich plasma is removed, 2 ml RBCs were removed and processed as previously described[1]^48^. Briefly, RBC samples were centrifuged 500*g* for 10 min at 4 °C. The top aqueous layer and white blood cells were removed and 1 ml RBCs from the bottom of the tube was diluted with 1 ml of RBC Wash Buffer (21.0 mM TRIZMA, 4.7 mM KCl, 2.0 mM CaCl2, 140.5 mM NaCl, 1.2 mM MgSO4, 5.5 mM glucose and 0.5% BSA at pH 7.4). Samples were centrifuged as before. The top aqueous layer was removed and the sample was diluted 1:1 with RBC Wash Buffer and centrifuged as before 2 more times. After the final centrifugation, the aqueous layer was removed and 100 μl RBCs were removed from the bottom of the tube for RNA isolation and flow cytometry analysis.

Platelets—Citrated whole blood was centrifuged at 150*g* for 17 min with no brake. Fifty per cent of the resulting platelet rich plasma was diluted 1:1 with platelet wash buffer (10 mM sodium citrate, 150 mM sodium chloride, 1 mM EDTA, 1% dextrose, pH 7.4, with 1:10,000 prostaglandin E1) and centrifuged at 300*g* for 4 min with no brake. Seventy-five per cent of the resulting supernatant was diluted again 1:1 with platelet wash buffer and centrifuged as before. Seventy-five per cent of the resulting supernatant is run through a 5 μm syringe filter (Millipore), followed by the same volume of platelet wash buffer. The platelets were pelleted by centrifuging at 740*g* for 17 min with low brake. The resulting pellet was used for RNA isolation and flow cytometry analysis.

*Plasma*. Citrated whole blood was centrifuged at 150*g* for 17 min with no brake. Two millilitres of platelet rich plasma was then centrifuged at 16,000*g* for 5 min. The supernatant was then serial centrifuged at 300*g* for 10 min, 2,000*g* for 20 min, and 10,000*g* for 30 min at 4 °C to remove cellular debris. The resulting supernatant was then used for RNA isolation and flow cytometry analysis, while another portion was used to isolate exosomes.

*Exosomes*. A portion of the plasma isolated above was used to isolate exosomes using magnetic beads from the ExoCap for Serum Plasma Kit (JSR Life Sciences) according to the manufacturer's instructions for qRT-PCR. RNA was isolated from these exosomes as well as analysed by flow cytometry.

*Peripheral blood mononuclear cells*. Citrated whole blood in a CPT Vacutainer tube (Becton Dickinson) was centrifuged at 1,800*g* for 35 min. The resulting cell layer was centrifuged at 288*g* for 17 min. The pellet was washed in 1 × PBS and centrifuged at 2,000*g* for 10 min two times. The final pellet was resuspended in 1 × PBS and run through a 5 μm filter, which is then washed and the eflux opening cleared. The filter was reversed and flushed with 1 × PBS. This process was repeated one more time and the resulting cell suspension was pelleted by centrifugation at 2,000*g* for 10 min. Cells were used for RNA isolation and flow cytometry analysis.

*Neutrophils*. Citrated whole blood collected in a CPT Vacutainer tube was centrifuged at 1,800*g* for 35 min. The resulting cell layer was treated with ammonium chloride for 10 min on ice to lyse RBCs. Neutrophils were then isolated through negative selection using the Human Neutrophil Enrichment Kit (StemCell Technologies) according to the manufacturer's instructions. The resulting pellet was used to isolated RNA and analysed by flow cytometry.

*T and B cells*. Citrated whole blood collected in a CPT Vacutainer tube was centrifuged at 1,800*g* for 35 min. The resulting cell layer was washed and centrifuged at 300*g* for 10 min 2 times. Each cell type was isolated through negative selection using the Pan B Cell Isolation Kit or Pan T Cell Isolation Kit (Miltenyi Biotec) according to the manufacturer's instructions. The resulting cell pellet was used to isolate RNA and analyse through flow cytometer.

*Flow cytometry*. The above mentioned samples were analysed by flow cytometry to determine purity using the flowing fluorescently conjugated antibodies and corresponding isotype controls (eBioscience): anti-human CD235a FITC (RBC marker), anti-human CD41a PerCp-eFluor 710 (platelet marker), anti-human CD56 APC (NK cell marker), anti-human CD14 APC (monocyte marker), anti-human CD15 FITC (granulocyte marker), anti-human CD16 PE-Cyanine7 (neutrophil marker), anti-human CD9 FITC (exosome marker), anti-human CD3 FITC (T cell marker) and anti-human CD19 PerCP-Cyanine5.5 (B cell marker). Samples were analysed using a FACSCaliber flow cytometer with Cell Quest Software (BD Bioscience).

*Blood cell isolation purity*. Each blood component—plasma, exosomes, platelets, PBMCs, RBCs, T Cells, B Cells and neutrophils—was isolated as described from six healthy donors (three males and three females; mean age of 41.67; 100% Caucasian). Flow cytometry was performed to verify the cell type isolated and identify any major contaminants. For exosomes, the sample was positive for CD9 (an exosome marker; *P*<0.001) and negative for platelets. For platelets, the sample was positive for CD41 (a platelet marker; *P*<0.001) and <5% positive for all other cell markers, except RBCs (11.1%±0.95). RBC sample was positive for CD235a (a RBC marker; *P*<0.05) and <5% positive for other cell markers. For PBMC sample, it was negative for RBCs but positive for platelets (55.9%±0.87); potentially platelets attached to PBMCs, however, although a large percentage, the relative abundance of platelet RNA (less than 1,000 fold), makes this contamination unlikely to significantly influence the gene expression results.

### Statistical methods for RNAseq

RNAseq data from the BIDMC validation cohort was screened for all exRNAs with an average sequence read of at least 1,000 reads per million (RPM>1 K) to compare with the RNAseq data generated from the FHS. Any exRNA identified in the FHS cohort with average RPM>1 K observed in the BIDMC cohort samples were considered overlapping. We report the percentage of overlapping piRNA and snoRNA to indicate general validity of the existence of these novel exRNA in plasma. To estimate the degree co-expression across cohorts, we calculated the using Spearman's rank correlation of the log-transformed sequence number of reads for overlapping piRNA and snoRNA.

### Statistical methods for Framingham Heart Study cohort study

We studied plasma samples for 2,822 eligible subjects from the FHS offspring 8 cohort. Fifty-nine (2%) of subjects were excluded because of lab errors (that is, inaccurate volume of plasma pipetted, *n*=31; poor protein precipitation performance, *n*=23; or potential contamination, *n*=5) resulting in 2,763 subjects for subsequent analysis. All statistical analyses were performed using STATA 13.0. Descriptive statistics are displayed as mean±s.d. for continuous variables and count (percentage) for categorical variables. The qRT-PCR results (Cq) values were normalized (ΔCq) by the global mean of expressed results to account for inter-individual sample differences. exRNAs not expressed, that is, failing to surpass the Cq within a set period, were first assigned the maximum Cq value (24 cycles) allowed according to our qRT-PCR procedures. Ordinary least-squares linear regression models were used to test for association with median age and sex and the normalized Cq value for each small-RNA assessed. To account for the number of statistical comparisons conducted for each of the 471 exRNAs assessed we employed FDR correction set at 5%.

We also estimated the association with the 10-year risk for CVD among individuals 30–74 years of age using the FHS Risk score. This sex-specific prediction score is generated from the following risk factors: age, diabetes, smoking, treated and untreated blood pressure, total cholesterol and HDL levels.

### Factor analysis

We conducted an exploratory principal factor analysis to identify the factor structure of the miRNAs measured. Assigning non-expressed miRNAs the maximum Cq of 24 in the normalization procedures could inflate the correlation between unexpressed miRNAs. To account for the non-expressed miRNA values (while maintaining some of the information contained the abundance level) we scored the ΔCq values into an ordered three level variable: 0=no expression; 1=low expression (ΔCq≥0; that is, less expressed than global mean); and 2=high expression (ΔCq<0; that is, more expressed than global mean). From these ordered-ΔCq we generated the Spearman's rank correlation matrix for principal factor analysis. The number of retained factors was determined by examination of the scree plot of the eigenvalues and considered varimax rotated factor loadings >0.4 or <−0.4 defining factor membership for each miRNA.

### Statistical methods for blood cell isolation

Expression of each exRNA type was compared in isolated blood cell components (*N*=6 for each cell type). Ordinary least-squares regression models were fit to compare mean Cq values in each cell component to plasma samples as the referent. ΔCq levels (that is, plasma-Cq—blood cell component Cq values) at *P*<0.05 were considered statistically significant. The total number of piRNA (*N*=97), snoRNA (*N*=43) and miRNA (*N*=331) were stratified into those that were not statistically significantly different from plasma, those that were statistically significantly upregulated in plasma, and those that were statistically significantly downregulated in plasma.

## Additional information

**Accession codes:** The complete RT-qPCR data has been deposited in dbGaP under the accession number phs000007.v27.p10. The RNA-seq data can be accessed at http://genboree.org/exRNA-atlas/exRNA-Grids.rhtml?grid=analysisTable under Jane Freedman.

**How to cite this article:** Freedman, J. E. *et al*. Diverse human extracellular RNAs are widely detected in human plasma. *Nat. Commun.* 7:11106 doi: 10.1038/ncomms11106 (2016).

## Supplementary Material

Supplementary Information Supplementary Figures 1-2 and Supplementary Tables 1-3

Supplementary Data 1 Complete List of miRNA, piRNA and snoRNAs Expressed by RNAseq in 40 Participants of the Framingham Heart Study

Supplementary Data 2 Association of exRNAs with Age and Sex (RT-qPCR)

Supplementary Data 3 Forty Participant of the Framingham Heart Study: RNAseq of Plasma. A listing of age and sex for the 40 participants who underwent plasma RNA sequencing.

## Figures and Tables

**Table 1:** exRNA factor structured in Framingham Heart Study cohort (*N*=2,763).

		**Rate expressed**	**Cq**	Targets*	Unique targets*
	***N***	**Mean±s.d. (range)**	**Mean±s.d. (range)**	**(*N*)**	**(*N*)**
*miRNA*					
F1	108	49.4±22.3 (11.9, 93.5)	19.2±1.5 (9.7, 20.9)	23,240	3,205
F2	67	4.4±2.0 (1.1, 8.4)	20.3±0.6 (16.8, 20.9)	18,898	2,718
F3	40	94.8±2.9 (84.2, 98.3)	17.9±1.6 (11.9, 19.9)	15,487	645
F4	17	68.6±21.9 (22.7, 94.3)	19.9±0.6 (18.8, 20.9)	13,928	156
None	100	34.2±24.0 (1.4, 95.3)	20.4±0.6 (17.3, 21,2)		
					
*piRNA*					
F1	17	53.2±22.4 (11.5, 90.8)	20.0±0.5 (19.1, 20.9)		
F2	14	0.3±0.1 (0.1, 0.5)	18.0±2.4 (13.3, 20.6)		
F3	12	4.0±3.0 (1.4, 11.1)	20.6±3.2 (20.1, 21.1)		
F4	7	51.9±20.1 (22.1, 77.3)	20.3±0.4 (19.6, 20.7)		
None	47	18.9±29.2 (0.4, 99.6)	20.4±1.3 (16.0, 21.3)		
					
*snoRNA*					
F1	13	4.6±2.0 (2.0, 8.3)	20.0±0.5 (19.0, 20.7)		
F2	9	29.3±18.4 (13.3, 65.9)	20.3±0.7 (18.6, 20.8)		
F3	8	67.8±22.9 (43.6, 95.0)	19.6±0.9 (18.3, 20.4)		
F4	6	17.3±9.0 (8.8, 30.6)	20.4±0.1 (20.2, 20.6)		
None	7	41.1±32.2 (9.6, 92.7)	20.3±0.7 (19.4, 20.9)		

^*^miRDB Version 5.0 target predictions available at (http://mirdb.org/miRDB/download.html).

**Table 2:** exRNA expression in each cell type relative to isolated plasma.

	**Not significant vs plasma**	**Upregulated in plasma**	**Downregulated in plasma**
	**(*N*)**	**(*N*)**	**(*N*)**
*piRNA (*N*=97)*			
B cells	62		35
Exosomes	89	7	1
Neutrophils	67		30
Peripheral mononuclear blood cells	49		
Platelets	49		48
Red blood cells	48		49
T cells	57		40
			
*snoRNA (*N*=43)*			
B cells	2		41
Exosomes	36	6	1
Neutrophils	6		37
Peripheral mononuclear blood cells	1		42
Platelets	22		21
Red blood cells	4		39
T cells			43
			
*miRNA (*N*=331)*			
B cells	165	3	163
Exosomes	258	73	
Neutrophils	282	10	39
Peripheral mononuclear blood cells	61	2	268
Platelets	66	3	262
Red blood cells	100	4	227
T cells	85	3	243

Noted are the number of exRNAs in each isolated cell type that were not statistically significantly different from plasma, that were statistically significantly enriched in plasma, and those that were statistically significantly depleted in plasma.
