# Corrigendum: Diverse human extracellular RNAs are widely detected in human plasma

**DOI:** 10.1038/ncomms11902

**Published:** 2016-06-03

**Authors:** Jane E. Freedman, Mark Gerstein, Eric Mick, Joel Rozowsky, Daniel Levy, Robert Kitchen, Saumya Das, Ravi Shah, Kirsty Danielson, Lea Beaulieu, Fabio C. P. Navarro, Yaoyu Wang, Timur R. Galeev, Alex Holman, Raymond Y. Kwong, Venkatesh Murthy, Selim E. Tanriverdi, Milka Koupenova, Ekaterina Mikhalev, Kahraman Tanriverdi

Nature Communications 7: Article number: 11106 10.1038/ncomms11106 (2016); Published: 04262016; Updated: 06032016

The original version of this Article contained an error in the spelling of the author Milka Koupenova, which was incorrectly given as Milka Koupenova-Zamor. This has now been corrected in both the PDF and HTML versions of the Article.

