# Supplementary material for: Diverse human extracellular RNAs are widely detected in human plasma
Source: Nat Commun. 2016 Apr 26;7:11106. doi: 10.1038/ncomms11106 (PMC4853467; doi:10.1038/ncomms11106)
Supplement: Supplementary Information — Supplementary Figures 1-2 and Supplementary Tables 1-3 [file ncomms11106-s1.pdf]

SUPPLEMENTARY FIGURES

A

Isolation Methods / Analysis Parameters	Exiqon Biofluid Kit (Exiqon=EX)	In-House Kit (In-House=IH)	TaqMan ABC Kit (LifeTech=LT)	Qiagen Serum/Plasma Kit (Qiagen=QI)
Mean Cq Values	18.20	17.25	17.27	19.17
Mean Standard Deviation	0.95	0.41	1.13	0.73
Mean CV	5.10	1.86	6.75	3.77

Three RNA isolation replicates were performed for each isolation kit from the same plasma sample. All RNA samples were analyzed for 90 miRNA assays (miScript miRNA assays from Qiagen) on the BioMark platform.

Exiqon Biofluid kit was chosen for further RNAseq and RT-qPCR analysis because of easier application and organic solvent free procedures.

RNA isolations from 2,763 plasma samples (Exiqon Biofluid Kit)

B

RNAseq

The aim of the RNAseq analysis was discovery of all possible targets in plasma samples. Target miRNAs, snoRNAs and piRNAs names were assembled and measured by RT-qPCR. The Ion Proton Sequencing platform was chosen because of its short running time as compare to the Illumina platforms. The sequencing was all completed in-house.

Library Preparation Method:
Ion Total RNA-Seq Kit v2 for small RNA kit used for library methods. This is the only kit deigned to work with Ion Torrent Proton sequencer.

RNAseq:
The PI Sequencing chips from Life Technologies and template preparation / emulsion PCR steps were performed in Ion Chef instrument automatically.

Adapter Removal:
Raw sequences were trimmed and cleaned from adapter and PCR primer sequences including sample barcode sequences on Ion Torrent servers connected to Proton sequencer.

Sequence Alignment and Analysis:
All alignment and further analyses were performed Yale University Medical School using the exceRpt Small RNA-seq Pipeline.

C

RT-qPCR

miScript Assay technology was chosen for all qPCR analyses. Two other technologies -TaqMan miRNA and Exiqon LNA Assays- were also considered and evaluated. TaqMan miRNA does not have universal cDNA reactions for all miRNA assays and their A and B panels were limited to 774 miRNAs. Exiqon LNA Assay technology did not have a standard preamplification protocols available which are mandatory when using the BioMark platform.

Reverse Transcription and Preamplification:
miScript II RT kit from Qiagen was been used for cDNA reactions. cDNA samples diluted 1:5 times and preamplified by using miScript Microfluidics PreAMP Kit from Qiagen and primer pool for miRNA assays.

qPCR Analysis:
All preamplified samples were diluted 1:5 times and qPCR experiments were performed with the Dynamic Array 96.9 on BioMark System from Fluidigm Inc.

Supplementary Figure 1. Mean Cq values, mean standard deviation and mean coefficient variation (CV) values of all 90 miRNA miScript Assays for all isolation kits (A). RNAseq analysis details (B). RT-qPCR details (C).

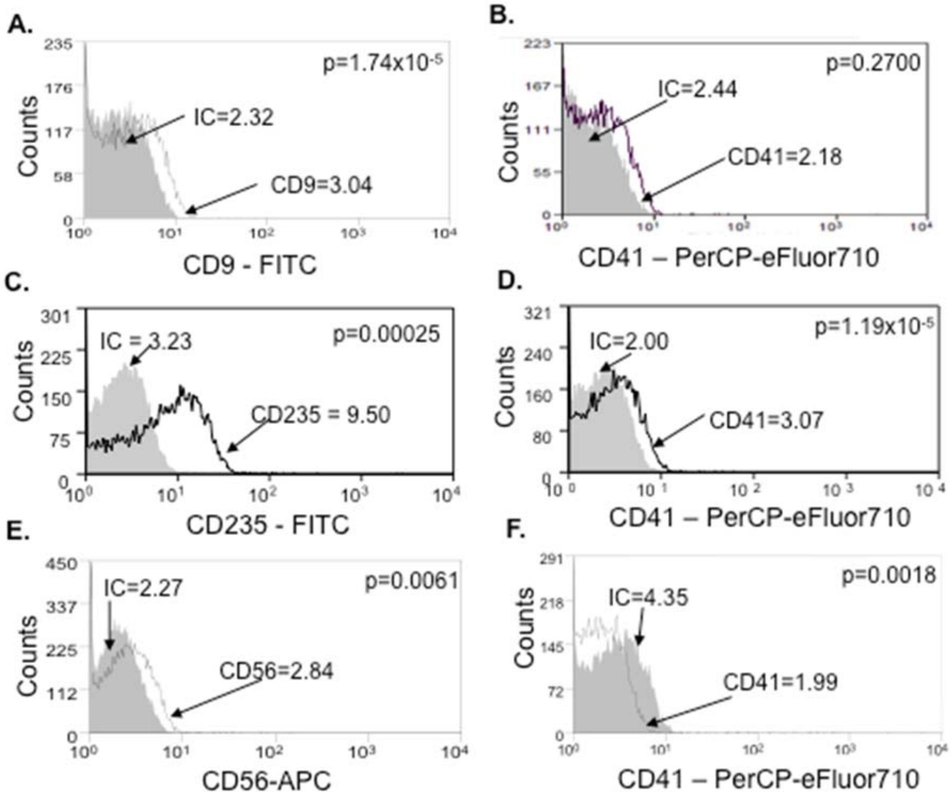
A.
p=1.74x10-5
IC=2.32
CD9=3.04
Counts
CD9 - FITC
B.
p=0.2700
IC=2.44
CD41=2.18
Counts
CD41 – PerCP-eFluor710
C.
IC = 3.23
p=0.00025
CD235 = 9.50
Counts
CD235 - FITC
D.
IC=2.00
p=1.19x10-5
CD41=3.07
Counts
CD41 – PerCP-eFluor710
E.
IC=2.27
p=0.0061
CD56=2.84
Counts
CD56-APC
F.
p=0.0018
IC=4.35
CD41=1.99
Counts
CD41 – PerCP-eFluor710

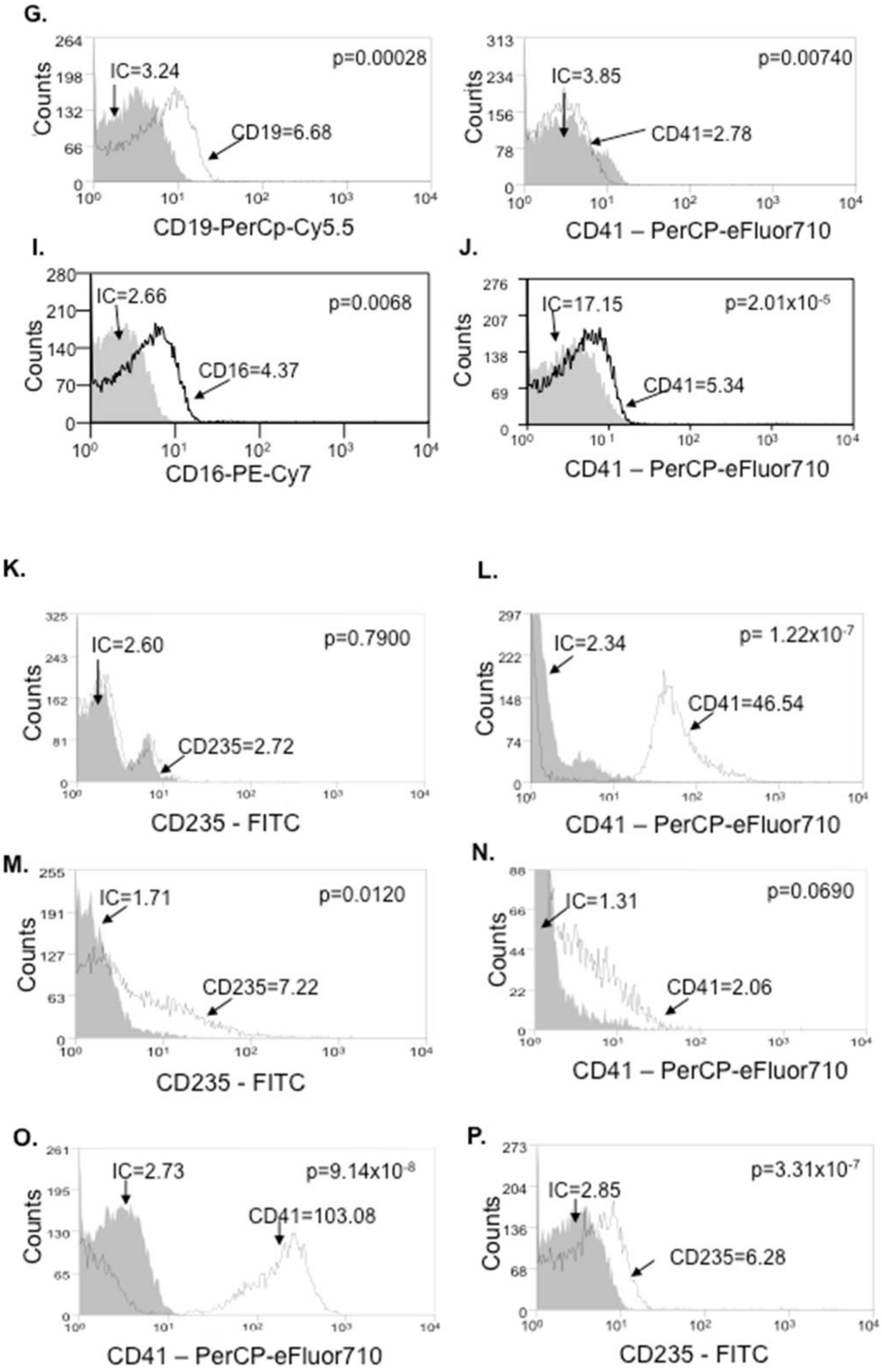

G.
Counts
IC=3.24
p=0.00028
CD19=6.68
CD19-PerCp-Cy5.5
Counts
IC=3.85
p=0.00740
CD41=2.78
CD41 – PerCP-eFluor710
I.
Counts
IC=2.66
p=0.0068
CD16=4.37
CD16-PE-Cy7
J.
Counts
IC=17.15
p=2.01x10-5
CD41=5.34
CD41 – PerCP-eFluor710
K.
Counts
IC=2.60
p=0.7900
CD235=2.72
CD235 - FITC
L.
Counts
IC=2.34
p= 1.22x10-7
CD41=46.54
CD41 – PerCP-eFluor710
M.
Counts
IC=1.71
p=0.0120
CD235=7.22
CD235 - FITC
N.
Counts
IC=1.31
p=0.0690
CD41=2.06
CD41 – PerCP-eFluor710
O.
Counts
IC=2.73
p=9.14x10-8
CD41=103.08
CD41 – PerCP-eFluor710
P.
Counts
IC=2.85
p=3.31x10-7
CD235=6.28
CD235 - FITC

Supplementary Figure 2. Isolated blood components: Representative figures. After each isolation, different blood components were stained and fixed with the listed antibodies. The expression of different markers was resolved by flow cytometry using FSC and SSC and the appropriate marker. The following populations were assessed as follows: exosomes were tested for **A.** exosomal marker and **B**. platelet-contamination; plasma was tested for **C.** red blood cell (RBC) marker and **D.** platelet contamination; T-cells were tested for **E.** T-cell marker and **F.** platelet contamination; B-cells were tested for **G.** B-cell marker and **H.** platelet contamination; Neutrophils were for **I.** neutrophil marker and **J.** platelet contamination; Peripheral blood mononuclear cell (PBMC) were tested for **K.** RBC and **L.** platelet contamination; RBC population was tested for **M.** RBC marker and **N.** platelet contamination; platelet population was tested for **O.** platelet marker and **P.** RBC-contamination. *p-values* were calculated based on two-tail t-test, using the isolations from at least three different donors.

Supplementary Table 1. High-throughput RT-qPCR of RNA Isolated from Plasma: Demographics of 2,763 Participants in the Framingham Heart Study Offspring Cohort (Visit 8).

**Clinical / Demographic Variables**	**Mean ± SD / N (%)**
Total N	2763
Age (years)	66.8±9.0
Sex (Female)	1499 (54%)
BMI	28.3±5.4
SBP	128.5±17.2
DBP	73.5±10.1
Triglycerides	118.4±69.6
Total:HDL Cholesterol	3.5±1.1
CVD	622 (23%)
Current Diabetes	383 (14%)
Current Smoker	247 (9%)
Antihypertensive Rx	1466 (53%)
Lipid Lowering Rx	1336 (48%)

Supplementary Table 2. Expression averages (reads per million) and numbers of expressed individuals of top expressed miRNAs , piRNAs and snoRNAs in human plasma samples.

	Exprs. Ave	N		Exprs. Ave	N		Exprs. Ave	N
hsa-miR-451a	328403.46	40	PIRPIR54042 27	2295.39	40	snoRNASNO1283 114	86.39	39
hsa-miR-223-3p	137957.11	40	PIRPIR2888 30	1684.62	40	snoRNASNO1550 138	45.40	39
hsa-miR-29b-3p	118459.88	40	PIRPIR58596 31	1604.31	40	snoRNASNO1209 144	37.22	39
hsa-miR-23a-3p	41116.77	40	PIRPIR43376 32	327.78	40	snoRNASNO1401 126	21.07	34
hsa-miR-19b-3p	31415.67	40	PIRPIR57581 31	312.29	39	snoRNASNO1404 75	19.87	31
hsa-miR-486-5p	17064.57	40	PIRPIR54043 26	59.41	40	snoRNASNO1408 217	19.47	29
hsa-miR-191-5p	14155.37	40	PIRPIR59288 32	57.53	33	snoRNASNO1212 255	16.79	34
hsa-miR-16-5p	10626.72	40	PIRPIR40304 32	44.88	40	snoRNASNO1405 72	15.29	23
hsa-miR-30d-5p	9933.22	40	PIRPIR41574 31	41.07	40	snoRNASNO1460 72	13.90	28
hsa-miR-21-5p	9069.71	40	PIRPIR227919 21	34.69	40	snoRNASNO1441 71	13.51	30
hsa-miR-222-3p	8767.63	40	PIRPIR75448 31	33.04	40	snoRNASNO1399 80	12.32	33
hsa-miR-19a-3p	8321.80	40	PIRPIR45809 31	28.77	34	snoRNASNO1466 136	12.28	30
hsa-miR-126-5p	7991.42	40	PIRPIR57849 32	26.67	20	snoRNASNO1532 152	10.72	31
hsa-miR-150-5p	7958.92	40	PIRPIR57322 27	20.90	36	snoRNASNO1568 138	10.12	31
hsa-miR-20a-5p	7019.79	40	PIRPIR59786 31	18.06	30	snoRNASNO1458 65	10.07	27
hsa-miR-126-3p	6832.52	40	PIRPIR37665 28	17.91	23	snoRNASNO1549 135	9.31	30
hsa-miR-15a-5p	6248.94	40	PIRPIR52755 30	17.16	35	snoRNASNO1414 71	8.65	23
hsa-miR-142-5p	6217.99	40	PIRPIR55478 30	17.05	18	snoRNASNO1374 71	7.57	23
hsa-miR-15b-5p	6080.55	40	PIRPIR33872 31	16.74	33	snoRNASNO1417 83	7.04	22
hsa-miR-199a-3p	5709.05	40	PIRPIR31112 30	15.99	34	snoRNASNO1291 76	6.79	17
hsa-miR-342-3p	5564.52	40	PIRPIR49916 28	15.85	31	snoRNASNO1382 70	5.96	17
hsa-miR-26a-5p	5265.23	40	PIRPIR59752 31	13.49	33	snoRNASNO1387 80	5.46	17
hsa-miR-101-3p	5141.78	40	PIRPIR59421 32	12.25	28	snoRNASNO1426 69	5.32	21
hsa-miR-140-3p	4475.59	40	PIRPIR51124 27	11.73	23	snoRNASNO1490 128	4.67	21
hsa-let-7b-5p	4418.95	40	PIRPIR1340 31	10.51	33	snoRNASNO1413 70	4.59	14
hsa-miR-103a-3p	4376.33	40	PIRPIR57576 29	9.72	1	snoRNASNO1467 78	4.42	21
hsa-miR-146a-5p	4181.49	40	PIRPIR248758 25	9.49	31	snoRNASNO1563 133	4.26	19
hsa-miR-484	4072.84	40	PIRPIR198666 28	8.77	12	snoRNASNO1257 74	3.89	14
hsa-miR-24-3p	3913.02	40	PIRPIR75519 28	8.73	2	snoRNASNO1502 130	3.54	19
hsa-miR-185-5p	3912.96	40	PIRPIR32637 32	7.80	21	snoRNASNO1562 137	3.45	19
hsa-miR-145-5p	3837.63	40	PIRPIR79118 29	7.34	23	snoRNASNO1277 77	3.45	13
hsa-miR-335-5p	3820.39	40	PIRPIR59027 31	6.75	20	snoRNASNO1425 71	3.26	12
hsa-miR-200c-3p	3523.42	40	PIRPIR37666 30	6.67	13	snoRNASNO1290 80	3.12	16
hsa-miR-29a-3p	3489.97	40	PIRPIR46511 28	6.21	18	snoRNASNO1507 133	2.89	10
hsa-miR-27a-3p	3393.08	40	PIRPIR266023 20	5.97	24	snoRNASNO1384 72	2.85	8
hsa-miR-30c-5p	3162.99	40	PIRPIR89449 30	5.12	24	snoRNASNO1465 85	2.69	9
hsa-miR-144-3p	2940.73	40	PIRPIR36598 30	4.56	19	snoRNASNO1472 93	2.51	11
hsa-miR-30e-5p	2878.41	40	PIRPIR37355 27	4.36	11	snoRNASNO1403 67	2.42	12
hsa-miR-92a-3p	2849.68	40	PIRPIR23216 29	4.27	19	snoRNASNO1407 65	2.34	12
hsa-miR-106b-5p	2826.76	40	PIRPIR75517 31	4.27	18	snoRNASNO1210 82	2.31	11

Supplementary Table 3. Expression (above one read per million) of identified exRNAs by RNAseq in 40 human participants.

**exRNA**	**Number of exRNAs**
Human miRNA	669
Human tRNA	305
Human piRNA	144
Human snoRNA	74
Total exRNAs	*1,192*
